# Effects of dexamethasone and gamma globulin combined with prednisone on the therapeutic effect and immune function of Henoch‐Schonlein purpura nephritis in children

**DOI:** 10.1002/jcla.23580

**Published:** 2020-11-11

**Authors:** Lei Chen, Xinning Wang, Liang Yin, Kun Ma, Xiangyang Liu

**Affiliations:** ^1^ Department of Pediatric Surgery Cangzhou Central Hospital Cangzhou China

**Keywords:** dexamethasone, gamma globulin, immune function, pediatric Henoch‐Schonlein purpura nephritis, prednisone

## Abstract

**Background:**

Henoch‐Schonlein purpura nephritis (HSPN) is a serious complication of Henoch‐Schonlein purpura (HSP), which is usually treated with immunosuppressant and glucocorticoid. This study was designed to explore the effect of dexamethasone and gamma globulin combined with prednisone in the treatment of pediatric HSPN.

**Methods:**

According to the treatment plan, 60 children treated with dexamethasone and gamma globulin were included in the control group, and the rest 55 children treated with dexamethasone and gamma globulin combined with prednisone were selected as the research group. The clinical manifestations, therapeutic effect, immune function, serum inflammatory factors, blood coagulation function, urine routine, renal function, and adverse reactions were compared between the two groups.

**Results:**

The clinical manifestations of children in the research group were significantly better than those in the control group after treatment (*P* < .05). The total effective rate in the research group (94.55%) was markedly higher than that in the control group (76.67%) (*P* < .05). CD3+, CD4+, CD8+, IL‐10, PT, and APTT increased while CD4+/CD8+, IgA, IL‐8, TNF‐α, FIB, urine protein, urine red blood cell, Scr, and BUN decreased in both groups after treatment, and the changes of all the above indexes in the research group were significant than those in the control group (*P* < .05). The incidence of adverse reactions in the research group was remarkably superior to that in the control group (*P* < .05).

**Conclusion:**

Dexamethasone and gamma globulin combined with prednisone can improve the immune function of children with HSPN and promote the recovery of renal function.

## Graphical abstract

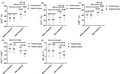

## 1 INTRODUCTION

Henoch‐Schonlein purpura (HSP) in children is a systemic inflammatory vascular disease with infection as the main inducement. In the initial stage, children with HSP often show extrarenal symptoms like skin purpura, joint pain, and hemorrhagic gastroenteritis and, in some cases, manifest as asymptomatic urine abnormalities. Generally, as the disease progresses, there will be renal injury of varying severity, glomerular damage, and interference with the reabsorption function of trace albumin.^1^, ^2^ While as a serious complication of HSP, Henoch‐Schonlein purpura nephritis (HSPN) is a kind of systemic disease with impaired renal parenchyma, which is more prevalent in children.^3^, ^4^ In most cases, it can be diagnosed by the occurrence of hematuria or proteinuria in the course of disease within 6 months in clinic.^5^ The pathological manifestations often change from necrotizing vasculitis to renal injury of varying degrees, persistent renal damage that eventually involves the whole body, and even renal failure.^6^ Today, the pathogenesis of HSPN has not been accurately determined, but it has been clinically confirmed to be related to the disorder of cellular and humoral immunity.^7^

Currently, immunosuppressive agents and glucocorticoid therapy for HSPN are widely used in clinical practice, but the treatment effect is still affected by factors such as poor tolerance and adverse reactions in children, leading to recurrence after drug withdrawal.^8^ Studies have shown^9^ that dexamethasone has a long‐lasting inhibitory effect on monocyte‐macrophages phagocytosis and destruction of antibody‐attached platelets, which can improve capillary permeability and peripheral blood circulation, and ensure the smooth hematopoiesis of bone marrow. While gamma globulin can prevent the formation of vicious circle of platelet damage by blocking Fc receptor reduction in mononuclear phagocytic system and reducing the production of autoantibody.^10^ However, at present, the combination therapy with the above two drugs cannot reach the satisfactory treatment effect in HSPN. As a new generation of anabolic steroids, prednisone has better anti‐inflammatory and anti‐immune biological efficacy than other glucocorticoids, but its application in HSPN still lacks relevant research. Therefore, this paper compared and analyzed the therapeutic effect of dexamethasone and gamma globulin combined with prednisone on children with HSPN, in order to find a better treatment for HSPN.

## 2 MATERIALS AND METHODS

### 2.1 General information

A total of 115 children with HSPN hospitalized in Cangzhou Central Hospital from July 2016 to August 2018 were selected as the research participants. Based on the treatment plan, 60 children treated with dexamethasone and gamma globulin were included in the control group, and the rest 55 children treated with dexamethasone and gamma globulin combined with prednisone were selected as the research group. Among them, there were 65 boys and 50 girls, and 24 cases of grade I, 54 cases of grade II, and 37 cases of grade III when classified by renal pathological grading, with an average age of 6.49 ± 1.42 years. There was no significant difference in age, gender, and other general information between the two groups (*P* > .05), suggesting comparability.

### 2.2 Inclusion and exclusion criteria

Inclusion criteria are as follows: Children who met the clinical diagnostic criteria of HSPN.^11^ Exclusion criteria are as follows: (a) Children treated with other hormones and immunosuppressants other than those used in this study within 1 week; (b) children with renal injury caused by viral hepatitis, vasculitis, lupus erythematosus, or other diseases; (c) children with nephrotic syndrome, IgA nephropathy, or other renal lesions; and (d) children with other system disorders. This experiment has been approved by the Medical Ethics Committee of Cangzhou Central Hospital and was conducted according to the international guidelines of Helsinki Declaration. The family members of the enrolled children understood the treatment details and signed the informed consent.

### 2.3 Treatment methods

Children in the control group were intravenously injected with dexamethasone (Huanan Pharmaceutical Group Co., Ltd., Guangdong, China, State Drug Approval Document Number: H44024469) at 0.25 mg/(kg·d), and gamma globulin (RAAS Blood Products Co., Ltd., Shanghai, China, State Drug Approval Document Number: SF20023011) at 400 mg/(kg·d). On this basis, the children in the research group received intravenous injection of prednisone (Xianju Pharmaceutical Co., Ltd., Zhejiang, China, State Drug Approval Document Number: H33021207] at 1 mg/(kg·d) 3 days later. Children in both groups were treated continuously for 1 month/course.

### 2.4 Outcome measures

(a) Clinical indicators such as the average renal involvement time, urinary protein excretion, and serum β2‐microglobulin were recorded by peripheral blood test and urine test. (b) The treatment efficacy was evaluated. According to clinical symptoms (complete disappearance, disappearance, improvement, no improvement, or exacerbation), 24 hours proteinuria, and urinary red blood cells (negative, decrease by more than 50%, decrease by more than 25%, no change), the treatment efficacy was divided into cured, improved, effective, and ineffective. Total clinical effective rate = (cured + improved + effective)/total number of cases × 100%.^12^ (c) The levels of immune function (CD3+, CD4+, CD8+, CD4+/CD8+, IgA) and serum inflammatory factors (IL‐8, IL‐10, TNF‐α) were measured and compared between the two groups. (d) The levels of coagulation function indexes (FIB, PT, APTT) were recorded and compared between the two groups. (e) The levels of urine routine indexes and renal function indexes (Scr, BUN) were detected and compared between the two groups. (f) The adverse reactions of the two groups were recorded and compared.

### 2.5 Statistical methods

Statistical analysis was performed using statistical software SPSS 21.0 (ND Times Technology Co., Ltd., Beijing, China). The counting data were compared by chi‐square test, while the measurement data were expressed as mean ± standard deviation, and intra‐ or inter‐group comparisons were performed by *t* test. *P* < .05 indicated a statistically significant difference.

## 3 RESULTS

### 3.1 Comparison of clinical manifestations between two groups of children with HSPN after treatment

After treatment, the average renal involvement time of (4.03 ± 0.67) weeks, urinary protein excretion of (347.35 ± 132.43) mg/d, and serum β2 microglobulin of (0.23 ± 0.07) mg/L) in the research group were dramatically better than those in the control group (*P* < .05). (Table 1).

**Table 1:** Comparison of post‐treatment clinical manifestations of children with Henoch‐Schonlein purpura nephritis receiving different treatment methods

Clinical manifestation	Control group (n = 60)	Research group (n = 55)	t	*P*
Average renal involvement time (weeks)	4.34 ± 0.73	4.03 ± 0.67	2.366	.020
Urinary protein excretion (mg/d)	552.63 ± 153.35	347.35 ± 132.43	7.651	<.001
Serum β2 microglobulin (mg/L)	0.49 ± 0.09	0.23 ± 0.07		

### 3.2 Comparison of therapeutic effects between the two groups of children treated by different treatment methods

The total effective rate in the research group was markedly higher than that in the control group (*P* < .05). (Table 2).

**Table 2:** Comparison of the therapeutic effect between the two groups of children treated by different treatment methods

Groups	Control group (n = 60)	Research group (n = 55)	X^2^	*P*
Cured	24 (40.00)	35 (63.64)	—	—
Improved	13 (21.67)	11 (20.00)	—	—
Effective	9 (15.00)	6 (10.91)	—	—
Ineffective	14 (23.33)	3 (5.45)	—	—
Total effective rate	46 (76.67)	52 (94.55)	13.461	<.001

### 3.3 Comparison of immune function between the two groups before and after treatment

**Figure 1:**
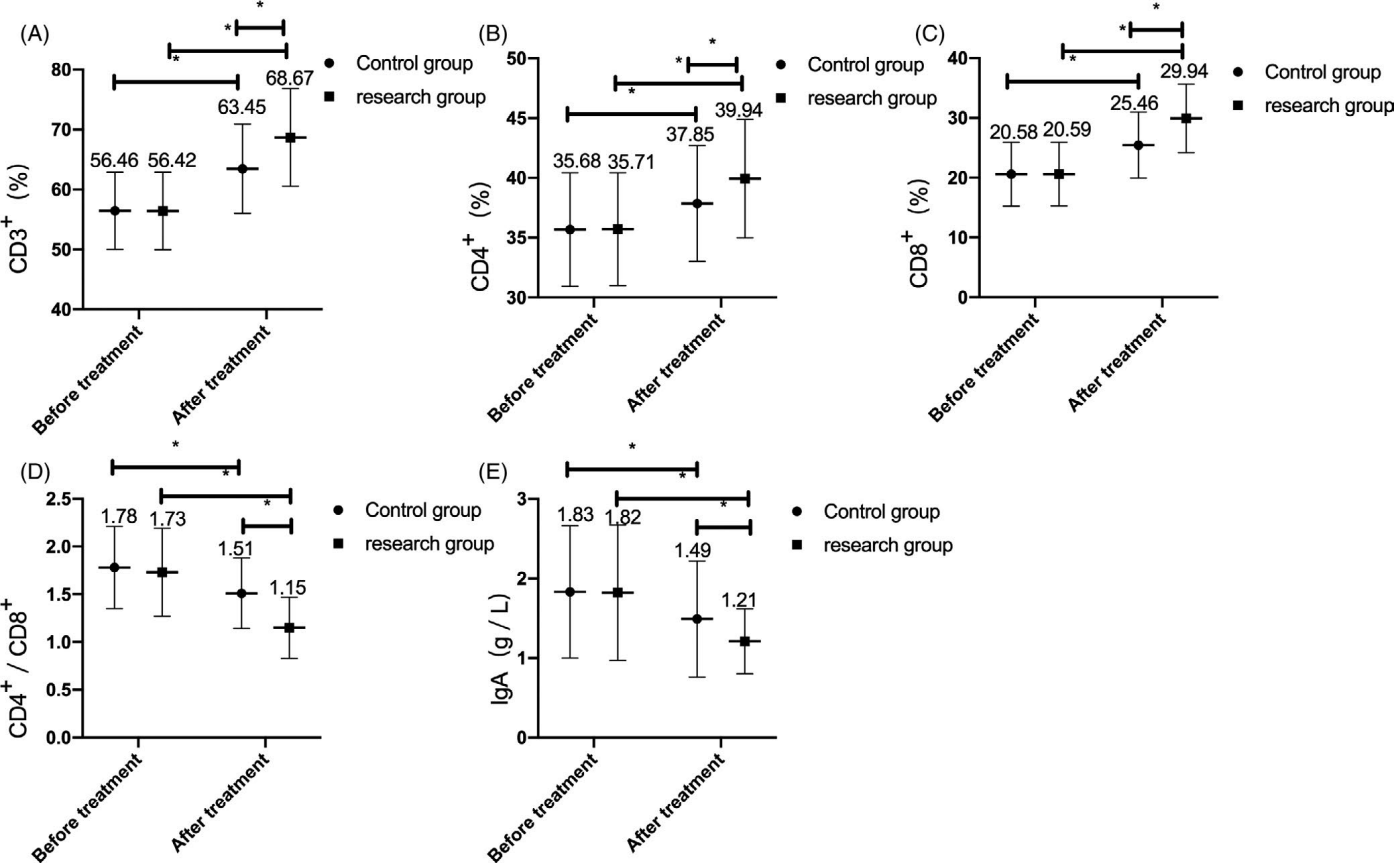
Comparison of immune function indexes between the two groups before and after treatment. A, After treatment, the CD3^+^ level of children in both groups increased, and its change in the research group was more obvious than that in the control group. B, After treatment, the CD4^+^ level of children in both groups elevated, and its change in the research group was more significant than that in the control group. C, After treatment, the CD8^+^ level of children in both groups increased, and its change in the research group was more significant than that in the control group. D, After treatment, the ratio of CD4+/CD8+ decreased in both groups, and the change in the research group was more significant than that in the control group. E, After treatment, the IgA level of the two groups of children declined, and the IgA level in the research group changed more significantly than that in the control group. Note: * represents the comparison between the two groups, *P* < .05

There were no significant differences in the immune function indexes of CD3+, CD4+, CD8+, CD4+/CD8+, and IgA between the two groups before treatment. After treatment, the levels of CD3+, CD4+, CD8+ increased, while CD4+/CD8+ and IgA decreased in the two groups, and the extent of change in the research group was more obvious than that in the control group (*P* < .05). (Figure 1).

### 3.4 Changes of serum inflammatory factors in the two groups before and after treatment

**Figure 2:**
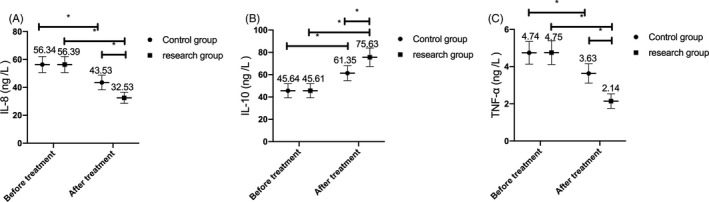
Comparison of the changes of serum inflammatory factors between the two groups before and after treatment. A, After treatment, the IL‐8 level of children in both groups decreased, and the change of IL‐8 level in the research group was more significant than that in the control group. B, After treatment, the IL‐10 level of children in both groups increased, and the change of IL‐10 level in the research group was more significant than that in the control group. C, After treatment, the TNF‐α level of children in both groups reduced, and the change of TNF‐α level in the research group was more significant than that in the control group. Note: * represents the comparison between the two groups, *P* < .05

No marked differences were noticed in serum inflammatory factors levels represented by IL‐8, IL‐10, and TNF‐α between the two groups before treatment (*P* > .05). After treatment, the levels of IL‐8 and TNF‐α in the two groups decreased, while IL‐10 level increased, and the change in the research group was more significant than that in the control group (*P* < .05). (Figure 2).

### 3.5 Changes of coagulation function in the two groups before and after treatment

**Figure 3:**
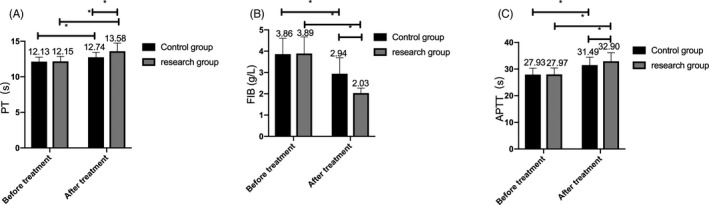
Changes of coagulation function in the two groups before and after treatment. A, After treatment, the PT increased in both groups, and the increase in the research group was more significant than that in the control group. B, After treatment, the FIB reduced in both groups, and the decrease in the research group was more significant than that in the control group. C, After treatment, the APTT elevated in both groups, and the increase in the research group was more significant than that in the control group. Note: * represents the comparison between the two groups, *P* < .05

Before treatment, there were no significant differences in coagulation function indexes between the two groups (*P* > .05). After treatment, FIB decreased while PT and APTT increased in the two groups, with a larger change extent in the research group (*P* < .05). (Figure 3).

### 3.6 Comparison of urine routine indexes between the two groups before and after treatment

**Figure 4:**
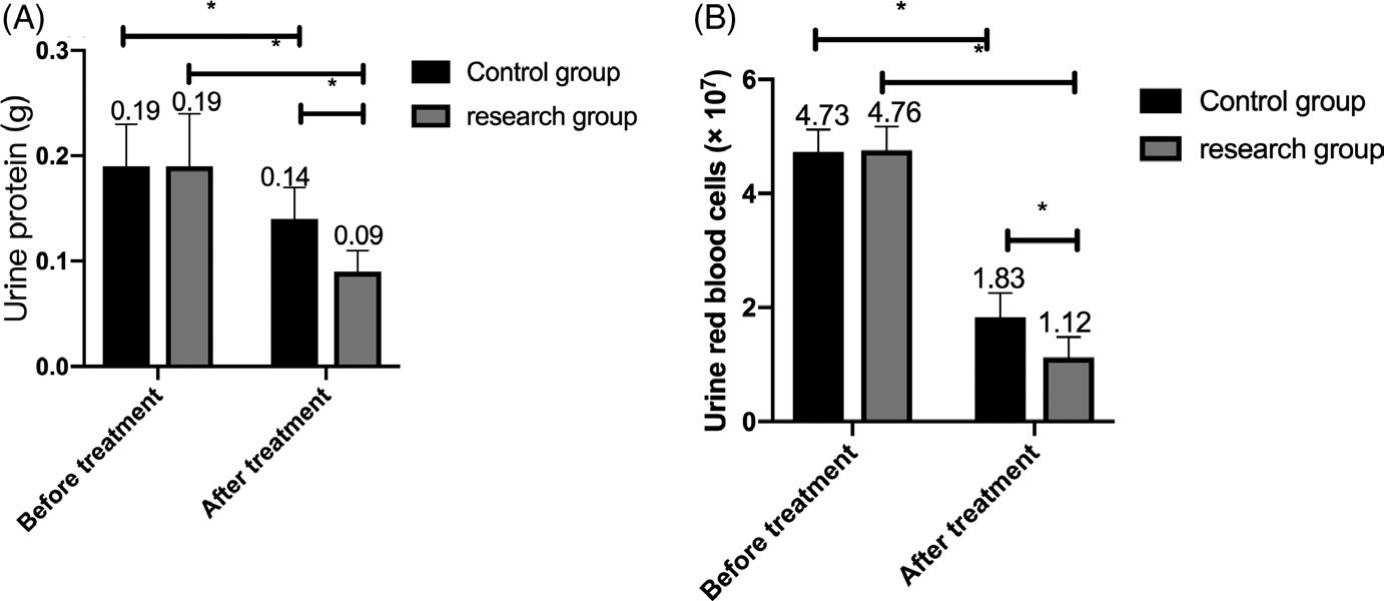
Comparison of urine routine indexes between the two groups before and after treatment. A, After treatment, the urine protein in both groups decreased within 24 h, and the change in the research group was more obvious than that in the control group. B, The urine red blood cells reduced in the two groups after treatment, and the decrease in the research group was more obvious than that in the control group. Note: * represents the comparison between the two groups, *P* < .05

The urine protein and urine red blood cell routine indexes did not identify any marked difference between the two groups before treatment (*P* > .05). While after treatment, the urine protein and urine red blood cell routine indexes dropped in both groups, and the change in the research group was more significant than that in the control group (*P* < .05). (Figure 4).

### 3.7 Comparison of renal function indexes between the two groups before and after treatment

**Figure 5:**
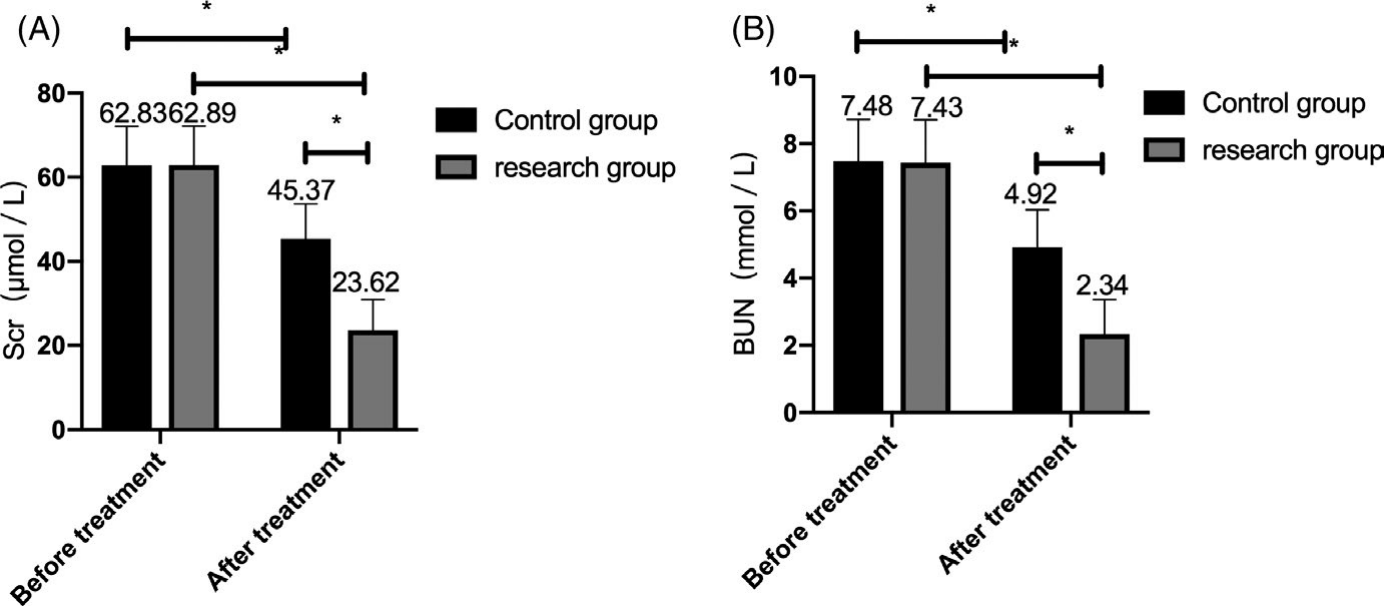
Comparison of renal function indexes between the two groups before and after treatment. A, After treatment, the Scr level decreased in the two groups, and the decrease in the research group was more obvious than that in the control group. B, After treatment, the BUN level reduced in both groups, and the decrease in the research group was more obvious than that in the control group. Note: * represents the comparison between the two groups, *P* < .05

The renal function indexes of Scr and BUN did not differ significantly between the two groups before treatment, but the Scr and BUN in both groups decreased after treatment, and the change in the research group was more obvious than that in the control group (*P* < .05). (Figure 5).

### 3.8 Comparison of adverse reactions between the two groups

The incidence of adverse reactions in the research group (9.09%) was remarkably lower than that in the control group (23.33%) (*P* < .05). (Table 3).

**Table 3:** Comparison of adverse reactions between the two groups [n (%)]

Groups	Control group (n = 60)	Research group (n = 55)	X^2^	*P*
Increased blood pressure	4 (6.67)	1 (1.82)	—	—
Myelosuppression	2 (3.33)	1 (1.82)	—	—
Infection	4 (6.67)	2 (3.64)	—	—
Edema	3 (5.00)	1 (1.82)	—	—
Palpitation	2 (3.33)	0	—	—
Total incidence of adverse reactions	14 (23.33)	5 (9.09)	7.292	.007

## 4 DISCUSSION

Henoch‐Schonlein purpura nephritis is a kind of vascular allergic immune complex disease, which can cause inflammation damage of small blood vessels and capillaries through IgA circulating immune complex stimulated by the common pathogenic bacteria Streptococcus.^13^, ^14^ During the process, large amounts of IgA complex deposition and Ig autoantibodies mediate mesangial cell antigens to aggravate glomerular endothelial cell damage and glomerular fibrosis.^15^ Clinically, HSPN is diagnosed through changes in skin, joints, gastrointestinal tract, and kidney manifestations, as well as the pathological changes of mesangial hyperplasia, which is similar to the pathogenesis of thrombocytopenic purpura and IgA nephropathy.^16^ For HSPN patients with severe lesions, although the formation of crescent at different degrees in vivo may relieve spontaneously, there may still be persistent proteinuria and renal failure.^17^ Therefore, effective diagnosis and intervention play a crucial role in improving the condition of patients with HSPN. Related studies have reported^18^, ^19^, ^20^ that there are many possible influencing factors (including age, rash status, gastrointestinal, and joint symptoms) for renal function impairment in HSP. As all the above factors can be analyzed from the aspects of inflammatory response and immunity, in this study, the combination of prednisone and the control drugs, dexamethasone, and gamma globulin were compared to observe the indicators influencing the improvement of prognosis in the two groups of children.

In vitro, the recovery degree of HSNP was compared mainly by objective phenomena such as urinary protein excretion and renal involvement time. The observation results showed that the research group was superior to the control group in clinical performance and treatment efficacy. In order to further explore the causes of different therapeutic effects, we specifically studied the changes in children's body during the treatment. It was found that there were no significant differences in inflammatory or immune indexes between the two groups of children before treatment, while the inflammatory and immune indexes were improved in both groups after treatment, and the extent of change in the research group was better than that in the control group. In HSPN patients, the interaction between CD40 of T cells and interleukin cells produced by Th2 cells can stimulate the activation, synthesis of autoantibodies, and mass proliferation of B cells.^21^ Dexamethasone can control the rate of platelet rise in the body and reduce the peak time of platelets, thus affecting the phagocytosis of immune cells in the body.^22^ As to gamma globulin, it contains broad‐spectrum IgG antibodies against bacteria or pathogens, and its immunoglobulin has a unique complex antibody immune network, which can produce certain anti‐infection and immunomodulatory effects. Its injection can improve the level of specific antibodies such as IgG in the body, inhibit Th2 from activating Th1, restore the balance between the two, and ease the allergic reaction of the body.^23^, ^24^ While by reducing the possibility of hyperemia, prednisone prevents inflammatory cells from moving toward the inflammatory site, controls the response of inflammatory mediators and phagocytosis, and regulates the stability of lysosomal membrane and Th factors.^25^ All these suggest that the drugs selected in this study can improve the immune function and reduce the occurrence of inflammatory transformation by regulating the immune system (media, complex) in children with HSPN, and the combination of prednisone can interfere with complement activation to improve the effect. Moreover, the coagulation function of the children was tested in this experiment. The results showed that the coagulation function of children in both groups improved after treatment, while that of the children in the research group improved better. Previous studies have reported^26^ that the free radical chain reaction caused by immune complex and complement activation in HSPN patients aggravates the degree of vascular endothelial depletion and that platelet parameters are proportional to the severity of the disease. This indicates that the combined use has a stronger regulating effect on the coagulation function of children and can interact with the immune ability and inflammatory regulation of children. In this study, the urine routine and renal function indexes verified that the combined use was more effective than the control group in improving renal function, and the combination of prednisone had strong ability to intervene and protect the immune function of children. Finally, the incidence of adverse reactions of each drug use was systematically observed, and the results revealed that the adverse reactions of children in the research group were less than those in the control group. The adverse drug reactions of children with HSPN after combined use did not increase, but decreased, which may be related to the stable regulation of immune function of prednisone.

To sum up, dexamethasone and gamma globulin combined with prednisone can enhance immune function and effectively promote the recovery of renal function in children with HSPN. However, there is still room for improvement in this study. To begin with, the insufficient sample size may lead to a large probability of error in data deviation, so we hope to increase the sample size in future research to reduce the deviation of results. In addition, in this study, literature review and comparison of experimental results were conducted merely on the influence of drugs on platelets, while the effect of drugs on children's other coagulation indexes was not elaborated or analyzed. Moreover, the reasons for the decrease of adverse drug reactions shall be further explored to better stabilize the efficacy of drugs. These are the directions of our follow‐up and improvement, so as to find a better treatment for this disease.

## References

[1] Xu H , Li W , Mao JH , Pan YX . Association between red blood cell distribution width and Henoch‐Schonlein purpura nephritis. Medicine (Baltimore). 2017;96(23):e7091. 28591051 10.1097/MD.0000000000007091 PMC5466229

[2] Zhao H , Huang WH , Huang JY , Lu SY , Yang YH , Ma ZG . Henoch‐Schonlein purpura nephritis associated with monoclonal gammopathy of renal significance: a case report. Braz J Med Biol Res. 2019;52(7):e8222. 31291381 10.1590/1414-431X20198222 PMC6694773

[3] Kuwabara T , Ohnishi T , Kakuta Y , Nomura S , Joh K . Successful treatment of allergic purpura nephritis associated with thrombotic microangiopathy using plasma exchange: a case report. Nihon Jinzo Gakkai Shi. 2004;46(8):815‐821. 15645739

[4] Ronkainen J , Ala‐Houhala M , Huttunen NP , et al. Outcome of Henoch‐Schoenlein nephritis with nephrotic‐range proteinuria. Clin Nephrol. 2003;60(2):80‐84. 12940608 10.5414/cnp60080

[5] Leung AK , Wong AH , Barg SS . Proteinuria in children: evaluation and differential diagnosis. Am Fam Physician. 2017;95(4):248‐254. 28290633

[6] Shah R , Ramakrishnan M , Vollmar A , Harrell A , Van Trump R , Masoud A . Henoch‐Schonlein purpura presenting as severe gastrointestinal and renal involvement with mixed outcomes in an adult patient. Cureus. 2017;9(3):e1088. 28405538 10.7759/cureus.1088 PMC5384843

[7] Popescu C , Leustean A , Orfanu AE , Carp CG , Arama V . Neutropenia and T‐Wave inversion as toxin‐mediated complications of a streptococcal infection. J Crit Care Med (Targu Mures). 2017;3(4):166‐171. 29967892 10.1515/jccm-2017-0030 PMC5769906

[8] Reynaud F , Giraud P , Cisterne JM , et al. Acute immuno‐allergic interstitial nephritis after treatment with fluindione. Seven cases. Nephrol Ther. 2009;5(4):292‐298. 19357010 10.1016/j.nephro.2009.01.008

[9] Standiford TJ , Kunkel SL , Rolfe MW , Evanoff HL , Allen RM , Strieter RM . Regulation of human alveolar macrophage‐ and blood monocyte‐derived interleukin‐8 by prostaglandin E2 and dexamethasone. Am J Respir Cell Mol Biol. 1992;6(1):75‐81. 1728298 10.1165/ajrcmb/6.1.75

[10] Saleh MN , Court WS , LoBuglio AF . In vitro effects of gammaglobulin (IgG) on human monocyte Fc receptor function. I. Effect on monocyte membrane‐associated IgG and Fc receptor‐dependent binding of antibody‐coated platelets. Am J Hematol. 1986;23(3):197‐207. 3094365 10.1002/ajh.2830230303

[11] Joh K , Shibasaki T , Azuma T , et al. Experimental drug‐induced allergic nephritis mediated by antihapten antibody. Int Arch Allergy Appl Immunol. 1989;88(3):337‐344. 2722256 10.1159/000234821

[12] Agrawal SR , Rajput A , Jain AP . Leukocytoclastic vasculitis and acute allergic interstitial nephritis following ceftriaxone exposure. J Pharmacol Pharmacother. 2014;5(4):268‐270. 25422573 10.4103/0976-500X.142453 PMC4231562

[13] Wang H , Xu J , Zhang X , et al. Tubular basement membrane immune complex deposition is associated with activity and progression of lupus nephritis: a large multicenter Chinese study. Lupus. 2018;27(4):545‐555. 28954590 10.1177/0961203317732407

[14] Delbet JD , Hogan J , Aoun B , et al. Clinical outcomes in children with Henoch‐Schonlein purpura nephritis without crescents. Pediatr Nephrol. 2017;32(7):1193‐1199. 28204946 10.1007/s00467-017-3604-9

[15] Hisano S , Matsushita M , Fujita T , Iwasaki H . Activation of the lectin complement pathway in Henoch‐Schonlein purpura nephritis. Am J Kidney Dis. 2005;45(2):295‐302. 15685507 10.1053/j.ajkd.2004.10.020

[16] Watanabe M , Fujimoto T , Iwano M , Shiiki H , Nakamura S . Report of a patient of primary Sjogren syndrome, IgA nephropathy and chronic idiopathic thrombocytopenic purpura. Nihon Rinsho Meneki Gakkai Kaishi. 2002;25(2):191‐198. 12043187 10.2177/jsci.25.191

[17] Kawasaki Y , Suzuki J , Nozawa R , et al. FB21, a monoclonal antibody that reacts with a sialic‐acid‐dependent carbohydrate epitope, is a marker for glomerular endothelial cell injury. Am J Kidney Dis. 2004;44(2):239‐249. 15264181 10.1053/j.ajkd.2004.04.028

[18] Kanashiki E , Nakazawa E , Akimoto T , et al. Case of Henoch‐Schonlein purpura nephritis complicated with essential mixed cryoglobulinemia. Nihon Jinzo Gakkai Shi. 2008;50(8):1024‐1029. 19172804

[19] Davol P , Mowad J , Mowad CM . Henoch‐Schonlein purpura presenting with orchitis: a case report and review of the literature. Cutis. 2006;77(2):89‐92. 16570670

[20] Eleftheriadis T , Liakopoulos V , Boulbou M , et al. Pulmonary renal syndrome in an adult patient with Henoch‐Shonlein purpura. Hippokratia. 2006;10(4):185‐187. 22087059 PMC2464257

[21] Hu X , Tai J , Qu Z , et al. A lower proportion of regulatory B cells in patients with Henoch‐Schoenlein purpura nephritis. PLoS One. 2016;11(3):e0152368. 27030970 10.1371/journal.pone.0152368 PMC4816555

[22] Walsh CJ , Wyffels JT , Bodine AB , Luer CA . Dexamethasone‐induced apoptosis in immune cells from peripheral circulation and lymphomyeloid tissues of juvenile clearnose skates, Raja eglanteria. Dev Comp Immunol. 2002;26(7):623‐633. 12074927 10.1016/s0145-305x(02)00016-2

[23] Goldman AS . 50 years ago in the Journal of Pediatrics: dysgammaglobulinemic antibody deficiency syndrome: increased gammaM‐globulins and Decreased gammaG‐ and gammaA‐globulins. J Pediatr. 2017;180:183. 28010792 10.1016/j.jpeds.2016.07.038

[24] Wolf HM , Thon V , Litzman J , Eibl MM . Detection of impaired IgG antibody formation facilitates the decision on early immunoglobulin replacement in hypogammaglobulinemic patients. Front Immunol. 2015;6:32. 25699049 10.3389/fimmu.2015.00032 PMC4313720

[25] Cutolo M , Hopp M , Liebscher S , Dasgupta B , Buttgereit F . Modified‐release prednisone for polymyalgia rheumatica: a multicentre, randomised, active‐controlled, double‐blind, parallel‐group study. RMD Open. 2017;3(1):e000426. 28405475 10.1136/rmdopen-2016-000426 PMC5372105

[26] Yuan Z , Yang X , Yuan Y . Relationship between platelet parameters on acute hypersensitive purpuric nephritis and kidney damage in children. J Bengbu Medical College. 2004;3:250‐251.

